# Investigating Virological, Immunological, and Pathological Avenues to Identify Potential Targets for Developing COVID-19 Treatment and Prevention Strategies

**DOI:** 10.3390/vaccines8030443

**Published:** 2020-08-06

**Authors:** Zafar Mahmood, Hani Alrefai, Helal F. Hetta, Hidaya A. Kader, Nayla Munawar, Sheikh Abdul Rahman, Shereen Elshaer, Gaber EI-Saber Batiha, Khalid Muhammad

**Affiliations:** 1 Lowance Center for Human Immunology, Department of Medicine, Emory University, Atlanta, GA 30322, USA; zafar.mahmood@emory.edu; 2 Department of Internal Medicine, University of Cincinnati College of Medicine, Cincinnati, OH 45267-0595, USA; alrefahd@ucmail.uc.edu (H.A.); helal.hetta@uc.edu (H.F.H.); 3 Medical Biochemistry Department, Mansoura Faculty of Medicine, Mansoura University, Mansoura 35516, Egypt; 4 Department of Medical Microbiology and Immunology, Faculty of Medicine, Assiut University, Assiut 71526, Egypt; 5 Department of Biology, College of Science, United Arab Emirates University, Al Ain 15551, UAE; 201790053@uaeu.ac.ae; 6 Department of Chemistry, College of Science, United Arab Emirates University, Al Ain 15551, UAE; nmunawar@uaeu.ac.ae; 7 Division of Microbiology and Immunology, Emory Vaccine Centre, Yerkes National Primate Research Centre, Emory University, Atlanta, GA 30322, USA; Sheikh.abdul.rahman@emory.edu; 8 Cincinnati Children’s Hospital Medical Center, Cincinnati, OH 45229-3026, USA; shereen.Elshaer@cchmc.org; 9 Public Health and Preventive Medicine Department, Faculty of Medicine, Mansoura University, Mansoura 35516, Egypt; 10 Department of Pharmacology and Therapeutics, Faculty of Veterinary Medicines, Damanhour University, Damanhour 22511, Egypt; gaberbatiha@gmail.com

**Keywords:** COVID-19, B cells, T cells, vaccine development

## Abstract

Severe acute respiratory syndrome coronavirus 2 (SARS-CoV-2) is an emerging coronavirus causing respiratory disease commonly known as COVID-19. This novel coronavirus transmits from human to human and has caused profound morbidity and mortality worldwide leading to the ongoing pandemic. Moreover, disease severity differs considerably from individual to individual. Investigating the virology of COVID-19 and immunological pathways underlying its clinical manifestations will enable the identification and design of effective vaccines and potential therapies. In this review, we explore COVID-19 virology, the contribution of the immune system (innate and adaptive) during infection and control of the virus. Finally, we highlight vaccine development and implications of immune system modulation for potential therapeutic interventions to design better therapeutic strategies to guide future cure.

## 1. Introduction

In late 2019, severe acute respiratory syndrome coronavirus 2 (SARS-CoV-2) was reported to have emerged in China, resulting in an unprecedented public health crisis. SARS-CoV-2 is the seventh identified human coronavirus, thought to have spilled over from bats into humans through an unknown intermediate [1,2]. Before the emergence of SARS-CoV-2, six human CoVs had been identified of which two are life threatening, SARS-CoV and Middle East respiratory syndrome coronavirus (MERS-CoV). SARS-CoV emerged in 2002 in China and ten years later, MERS-CoV was identified in the Middle East [3,4,5]. Currently, a third novel pathogenic CoV (2019-nCoV or SARS-CoV-2) has emerged, developing into a world public health emergency [6,7]. The name of this new emergent CoV reflects its high phylogenetic similarity to SARS-CoV [8]. SARS-CoV-2 causes coronavirus disease 2019 (COVID-19) and it was declared a pandemic viral outbreak by the World Health Organization (WHO) in March 2020 [9]. As of 26 July 2020, this pandemic has globally caused around ~15.5 million confirmed cases leading to around ~650,000 deaths to date [10]. Most patients exhibit mild to moderate symptoms but approximately 15–18% patients develop acute respiratory distress syndrome (ARDS), cytokine storm and/or multiple organ failure [11].

## 2. Coronaviruses, Origin and Genome Structure

**Figure 1:**
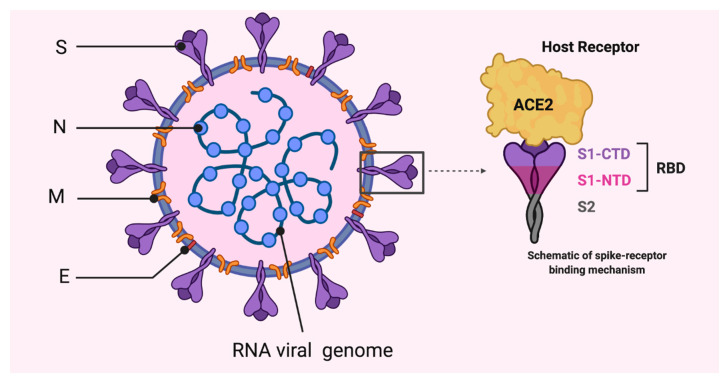
Coronavirus structure showing the organization of spike (S), membrane (M), and envelope (E) proteins. The viral RNA is associated with the nucleocapsid protein (N). ACE: angiotensin converting enzyme; RBD: receptor-binding domains; S1-NTD: N-terminal domain; S1-CTD: C-terminal domain; S1: amino termini; S2: carboxy termini.

SARS-CoV-2 belongs to the subgenus *Sarbecovirus* of the beta coronavirus genus, and subfamily *Orthocoronavirinae* of the *Coronaviridae* family [12]. It was recognized for the first time in December 2019 in Wuhan, a central city in China. There are four common human CoVs with low pathogenicity, resulting in mild diseases; two alpha-CoVs (229E and NL63) and two beta-CoVs (OC43 and HKU1) [1]. The previous pathogenic CoVs, SARS-CoVs and MERS-CoVs caused severe and potentially fatal respiratory tract infection in human populations [13]. SARS-CoV-2 belongs to beta-CoVs, which includes SARS- and MERS-related CoVs [14,15]. Like other CoVs, SARS-CoV-2 is an enveloped spherical particle (60–140 nm) with spike proteins on the surface (corona) (Figure 1) [12,14].

**Figure 2:**
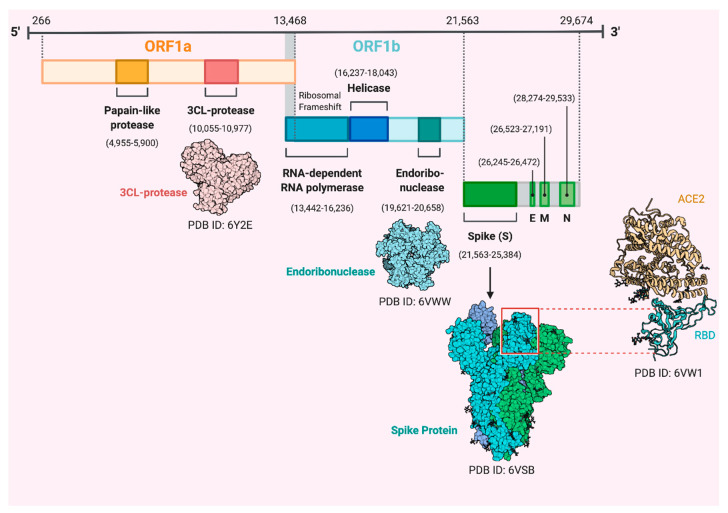
Genomic organization of severe acute respiratory syndrome coronavirus 2 (SARS-CoV-2), including open reading frames (ORF1a and ORF1b), spike (S), envelope (E), membrane (M), and nucleocapsid (N) proteins. Three-dimensional protein structures of 3CL-protease, endoribonuclease and spike protein bound to the human angiotensin converting enzyme 2 (ACE2) receptor are illustrated. PDB: Protein database.

The SARS-CoV-2 genome has 96.2% sequence identity to the bat CoV RaTG13 and it is 79.5% identical to SARS-CoV. Accordingly, bats are considered the natural host and a potential origin of the virus, and it may have transmitted to humans through an unknown intermediate [11,16] or directly from the wet wild animal market in Wuhan. SARS-CoV-2 is an enveloped virus with a single-stranded, positive-sense RNA genome (~30,000 nucleotides) having a 5′ cap structure and 3′ poly-A tail. It encodes several open reading frames (ORFs) [11,17,18,19,20,21]. Two-thirds of viral genome, mainly located in the first ORF (ORF1a/b), translates two polyproteins, pp1a and pp1ab, composed of 16 non-structural proteins (NSP1-NSP16), while the remaining ORFs, which are located near the 3′- terminus, encode accessory and structural proteins (Figure 2) [22,23]. The four main structural proteins are envelope protein (E), nucleocapsid protein (N), membrane protein (M) and spike (S) surface proteins [24,25,26]. The trimeric S protein, a type-I transmembrane glycoprotein on the surface of the virus that forms the corona, is composed of two domains, the amino (S1) and carboxy (S2) termini [25]. The two domains of S1 subunit, N-terminal domain “S1-NTD” and C-terminal domain “S1-CTD” compose receptor-binding domains (RBD) (Figure 1), determine the virus–host range and cellular tropism. The S2 subunit contains heptad repeat 1 (HR1) and heptad repeat 2 (HR2) domains, which mediate the virus–cell membrane fusion [27,28,29].

## 3. Replication and Pathogenesis

**Figure 3:**
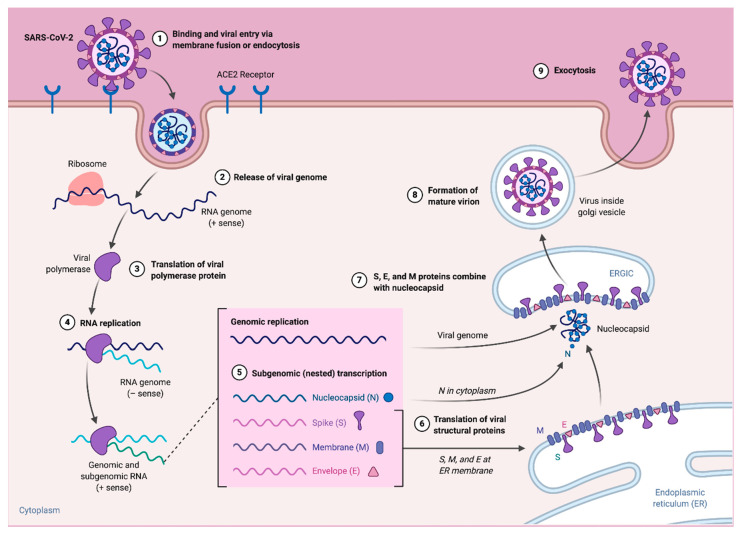
SARS-CoV-2 replication cycle and potential therapeutic targets.

The first step of CoVs replication is recognition and binding to host cell receptors via S1 subunit (Figure 3). SARS-CoV-2 binds to the same cellular entry receptor angiotensin converting enzyme 2 (ACE2) as SARS-CoV [16]. After binding with the ACE2 receptor, proteolytic cleavage of S protein occurs within the S2 domains by transmembrane protease serine 2 (TMPRRS2), or cathepsin, resulting in the virion membrane fusion with the host cell membrane [24]. This fusion releases the viral RNA into the host cytosol where translation of the two polyproteins, pp1a and pp1ab, begins and sixteen proteins are cleaved out of these proteins by various viral and host proteases releasing the non-structural proteins. Most of these non-structural proteins assemble to form the viral replicase–transcriptase complex (RTC) in double-membrane vesicles for subsequent synthesis of the nested set of subgenomic RNAs (sgRNAs), which encode structural and accessory proteins (Figure 3) [24,30]. Genomic and sgRNAs are produced through negative-strand intermediates. The genomic RNA and virion N protein along with the E, S and M proteins in the virion membrane are transported to ER-Golgi intermediate compartment (ERGIC) and assemble together to produce the virus particle. Finally, virus-containing vesicle fuses with the host cell membrane to be released by exocytosis [11,24]. Several therapeutic targets have been identified in the viral replication steps as indicated in the Figure 3 and are being explored for possible antiviral drug control strategies.

Pathogenesis of SARS-CoV-2 starts through the S1 domain, followed by cell membrane fusion mediated by S2 domain. ACE2 receptors are widely distributed in human cells lining the surface of the lungs, heart, kidney and liver although variation in expression levels between tissue and individuals is observed [16,31,32,33,34]. This distribution may contribute to the variable clinical manifestation of the disease and may explain why patients suffer not just from ARDS, but also acute myocardial and kidney injury, shock and death because of multiple organ dysfunction [35,36].

Diffuse pulmonary intravascular coagulopathy associated with SARS-CoV-2 infection is linked to the extensive alveolar and interstitial inflammation, which is attributed to macrophage activation syndrome (MAS)-like state, inducing extensive immunothrombosis [37,38].

The immune system is responsible for the resolution of COVID-19, however, overactivation of the immune system against SARS-CoV-2 can result in a severe cytokine storm due to the release of huge numbers of inflammatory factors such as IL-2, IL-6, IL-7, GM-CSF, IP10, MCP1, MIP1A, and TNF-α [35,36]. This virus-induced cytokine storm may be a cause of organ dysfunction and damage [35]. Understanding key targets in suppressing hyper immune activation may help improve disease severity and progression therefore representing a key avenue of exploration for possible treatment strategies.

## 4. Clinical Characterization

**Figure 4:**
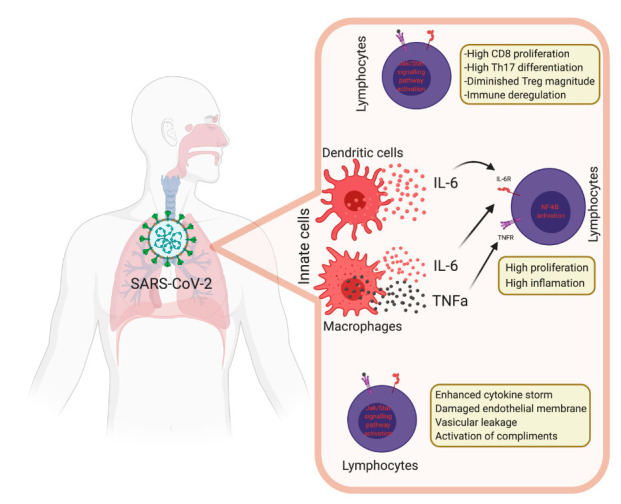
Immune dysregulation mechanisms during coronavirus disease 2019 (COVID-19).

SARS-CoV-2 is associated with human-to-human transmission and is thought to spread by sneezing and respiratory droplets formed by coughing and by direct fomite transmission [39]. Additionally, isolation of SARS-CoV-2 from blood and fecal swabs suggests multiple transmission routes [40]. SARS-CoV-2 airborne transmission may be possible in specific conditions. Due to its persistence in aerosol droplets, a viable and infectious form can be possible over longer periods of time [41,42]. Once infection is established, the clinical spectrum of COVID-19 varies from asymptomatic to multi-organ failure. The symptoms of mild disease include fever, cough, fatigue, dyspnea, headache and sore throat but in severe cases, the disease worsens 5–10 days after onset of infection. The incubation period for symptomatic mild patients, the time from exposure to symptom onset, is 4–5 days on average [43,44,45]. Some patients may have gastrointestinal symptoms such as vomiting and diarrhea. However, in severe cases, individuals with COVID-19 develop signs and symptoms of ARDS, which requires mechanical ventilation [46]. Prediction of COVID-19 severity and diseases risk are complicated. Routine bloodwork often finds low lymphocyte counts, high C-reactive Protein (CRP) and lactate dehydrogenase (LDH) levels and a high ratio of platelet/lymphocyte, which is positively correlated with the disease severity [47,48]. Thrombocytopenia is associated with incidence of myocardial injury in COVID-19 and coagulation parameters predict severity independently of other variables [49,50]. Moreover, prognostic immune biomarkers in COVID -19 patients were identified with the disease severity. Peripheral blood analysis demonstrates significantly lower counts of CD4+ T, CD8+ T, and NK cells in PBMCs and increased expression of Programmed cell death protein 1 (PD-1) and Tim-3 on T cells and has been correlated with severity of COVID-19. There is likely initial delay in the antiviral response and subsequent increased production of inflammatory cytokines with an influx of monocytes and neutrophils into the lung, leading to the cytokine storm syndrome [44]. There are also phenotypic changes in peripheral blood monocytes with a distinct population which secrete IL-6, IL-10, and TNF-α. IL-4, IL-6, IL-8, IL-10, IL-18, IL-2, IFN-γ, IL-2R and IL-1β along with other cytokines showing a correlation with disease severity. CD14+CD16+GM-CSF+ monocytes are higher in COVID-19 patients than healthy and GM-CSF+IFN-γ+ T cells are higher in ICU patients than in non-ICU patients [51]. Single-cell RNA-seq analysis in early-recovery patients has identified abundant CD14+IL-1β+ monocytes, thought to be associated with the cytokine storm [52]. T cell counts and cytokine levels (Figure 4) in severe COVID-19 patients who survive the disease gradually recover at later time points to levels which are comparable to mild infection. As all patients are lymphopenic, the neutrophil-to-CD8+ T cell ratio, counts of CD4 and CD8 T cells, and cytokine IL-6 and IL-10 can be identified as the most powerful prognostic factors for the progression of disease from mild to severe CoV-2 infection [53]. Additionally, computed tomography on the chest shows ground-glass opacity and bilateral patchy shadows [54].

## 5. Innate Immunity during COVID-19

The innate immune system of the host is the first to react with the viral infections helping to detect and control them. This detection is achieved by using pattern recognition receptors (PRRs) such as toll-like receptors (TLRs), NOD-like receptors (NLRs), RIG-I-like receptors (RLRs) and C-type lectin-like receptors [55,56]. PPRs recognize pathogen-associated molecular patterns (PAMPs) such as lipo-proteins, lipids, nucleic acids, and proteins of viral origin in the host cell membranes including lysosomes and endosomes. Upon recognition, different biological responses are induced through the activation of varied adapter proteins leading to the release of pro-inflammatory cytokines that recruit other immune cells to the site of infection [57,58], inhibit viral replication and activate the adaptive immune response by pathogen-derived antigen presentation via activated dendritic cells (DCs) to naïve helper T cells. Natural killer cells (NKs) kill the infected cells through mechanisms such as degranulation, antibody-dependent cellular cytotoxicity and receptor mediated apoptosis. Macrophages and neutrophils eliminate the pathogen and infected cells through phagocytosis. Even though all these mechanisms are important for viral elimination, an over-reactive immune response may contribute to disease pathogenesis [59,60,61].

There is compelling evidence for innate immune hyperactivity during acute lung injury that is a hallmark of SARS-CoV-2 infections. Reduced blood lymphocytes and higher neutrophil counts are a hallmark of SARS-CoV-2. Neutrophils are recruited at the site of infection where they kill pathogens by oxidative burst and formation of neutrophils extracellular traps (NETs) [62]. SARS-CoV-2 infection and lung cell destruction trigger a local immune response by recruiting macrophages and monocytes that release cytokines and prime adaptive immune response [63]. A dysfunctional immune response can cause severe lung and systemic pathology inducing injury and death of virus infected cells and tissues. Tissue-resident macrophages are involved in the epithelial damage during the acute respiratory distress syndrome [64].

Macrophages are activated after detecting the PAMPs in viral RNA by their pattern recognition receptor as well as damage associated molecular patterns (DAMPs) including ATP, DNA and antibody-secreting cell (ASC) protein oligomers [60,65]. These DAMPs and PAMPs are likely generated during SARS-CoV-2 infection leading to lysis of pneumocytes, resulting in a wave of local inflammation and secretion of proinflammatory cytokines including IL-6, IL-1β and IFN-γ as well the chemokines IP10 and MCP1 into the blood of infected patients [34] leading to other cytokine production and antiviral gene activation, especially interferon-stimulated genes uniquely upregulated in NK cells. The inflammatory cascade triggered by macrophages leads to viral control, through production of interferon I and III, thereby promoting antiviral defenses in neighboring epithelial cells, and tissue damage. The secretion of cytokines, mainly IL-6 and IL-1β, also promotes the recruitment of other immune cells, mainly monocytes and T lymphocytes, to the infection sites. Such infiltration of lymphocytes to the infected airways might explain the lymphopenia in the majority of SARS-CoV-2 infected patients [54,66]. Recent studies have shown elevated IL-6 levels in COVID-19 non-survivors compared to survivors [33]. Increased inflammatory monocyte-derived FCN1+ macrophages have been observed in bronchioalveolar lavage fluid from severely ill patients compared with mild COVID-19 patients. Similarly, an increased population of CD14+CD16+ inflammatory monocytes cells have been observed in severely diseased patients indicating a significant contribution of innate immune cells in COVID-19 disease [67]. Wilk et al. reported that in CD14+ monocytes, human leukocyte antigen (HLA) class II expressions, as well as a pathway associated with DC–NK cell immune crosstalk, were reduced, whereas a pathway associated with PD1–PDL1 interactions was increased. Collectively, these results indicate that dysregulation of immune crosstalk is associated with severity of COVID-19. Furthermore, a functional exhaustion of natural killer (NK) cells and cytotoxic T cells (CTLs), which are required for an effective antiviral immune response, enhances disease progress [67]. This exhaustion is believed to be triggered by the overexpression of a prominent heterodimeric NK inhibitory receptor, namely, NK group-2 member A (NKG2A) that is overexpressed in CD8^+^ and NK cells [68]. NKG2A transduces inhibitory signals on ligation with peptide loaded HLA-E thereby suppressing cytokine secretion by NK and its cytotoxicity. This results in overriding the innate immune response of the host by SARS-CoV-2 and also increases the opportunities of co-infections of the lung in patients with severe symptoms [69].

## 6. Adaptive Immunity during COVID-19

### 6.1. T Cell Immune Response to SARS-CoV-2 Infection

T lymphocytes, CD4+ T cells and CD8+ T cells, play a vital role in balancing their antiviral activity with the possibility of developing an excessive immune inflammatory response or autoimmunity [70,71,72]. While CD8+ T cells help in direct elimination of virally infected cells through cytotoxicity, CD4+ T cells are vital for stimulating CD8+ T cells and B cells. In SARS-CoV-2 infection, it is uncertain if the adaptive immune response is helpful or harmful in combating the condition. A late T cell response may contribute towards worsening the disease condition through amplification of the pathogenic inflammatory response in the presence of huge viral loads in the lungs [73]. Furthermore, previous studies on SARS-CoV infections have indicated that at the time of infection, a depletion in CD8+ T cells does not affect or delay the viral replication. However, depletion in CD4+ T cells during the infection results in a delayed viral clearance accompanied by strong immune-mediated interstitial pneumonia that is triggered by reduced neutralizing antibodies, recruitment of pulmonary lymphocytes and cytokine production [74]. Moreover, monocytes and neutrophils are recruited to the site of infection by the IL-17 released by T helper cells, which further exacerbates the condition via release of downstream cytokines and chemokines, such as TNFα, MCP-1, IL-6, IL-1, IL-8 and IL-21 [60].

Studies on autopsy and blood samples of COVID-19 patients have revealed that the peripheral blood contains a low number of hyperactive T lymphocytes. Most of the T lymphocytes had accumulated in the lungs, suggesting an infiltration of T cells from blood to the site of infection [45]. The SARS-CoV-2 patients exhibit a Th1 mediated immune response where TNF, INF-γ and IL-2 are expressed by SARS-CoV-2 specific CD4+ T cells aiding in disease control by cell mediated immunity. A study in a mouse model, immunized with dendritic cells bearing SARS-CoV peptides resulted in the accumulation of large numbers of virus specific CD4+ and CD8+ T cells in the lungs [75]. This finding supported the possibility that the delay in T cell response during infection might be due to the lack of availability or function of antigen presenting cells (APCs). However, even though T cell responses controlled SARS-CoV infections, several protein vaccine formulations tested so far have resulted in immunopathological reactions, especially in aged animals via a Th2 mediated eosinophil infiltration [63]. Hence, more studies are required to understand the protective versus pathological T cell responses for vaccine formulations.

### 6.2. Role of Memory T Cell against SARS-CoV-2

Recent studies have investigated the existence of SARS-CoV-2 specific memory T cells [76,77] and SARS-CoV-2 specific CD4+ and CD8+ T cells, which were present in 80% of patients admitted to the ICU. The CD4+ T cells were predominately Th1 cells. Interestingly, 20% of the control subjects also showed SARS-CoV-2 specific T cells. The reactivity of the T cells in the patients and the controls was directed mainly toward the spike glycoprotein [78]. The researchers point out that there should be cross reactivity between common cold coronavirus and SARS-CoV-2 due to presence of memory T cells in uninfected individuals [78,79]. Another study showed that CD4+ T cells were found in 100% of the convalescent patients while CD8+ T cells were found only in 70% of the convalescent patients. The CD4+ T cells were reactive toward M, spike, and N proteins, which are different from SARS-CoV-1 where memory CD4+ T cells were reactive mainly toward spike protein. This difference could be important for SARS-CoV-2 vaccine development. Incorporation of SARS-CoV-2 structural antigens in addition to the spike protein might better mimic the efficient immune response in COVID-19 patients showing mild and moderate disease course. Similarly, an efficient vaccine that relies on eliciting CD8+ T cell response towards SARS-CoV-2 should make use of HLA class I epitopes derived from the M, nsp6, ORF3a, and/or N proteins [73].

### 6.3. B Cell Immunity during SARS-CoV-2 Infection

The B lymphocytes in the blood are involved in early effect or responses via producing protective antibodies in response to the initial challenge, as well as in initiating the production of memory cells to provide comprehensive immunity against repeated infection [80]. The B cell response to an infection, virus or vaccination, generates virus antigen-specific antibodies (Abs) that play a key role in providing a protective barrier to infection and in facilitating viral clearance. Both antibody-secreting cells (ASCs) and memory B cells are the product of antigen activation. Interaction of B cells with cognate T helper cells is the critical requirement in most cases for optimal antibody responses. This T cell help is important for directing antibody isotype switching in B cells, the process by which B cells switch from expressing IgM to expressing alternative isotypes IgGs and IgA with different functional characteristics [81]. ASCs can be divided into short-lived ASCs, including short-lived plasma cells, plasmablasts, and long-lived plasma cells. Plasmablasts are considered a transient population and can be either precursors of plasma cells (short- and/or long-lived; mainly in mice) or terminally differentiated effector cells. One of the hallmarks of the immune system is the memory of past exposure to infections (antigens). Thus, the success of vaccines is dependent on the generation and maintenance of long-term immunological memory.

There is great interest in understanding the B cell memory humoral responses to SARS-CoV-2 infection, which has been studied in the context of antibody distribution in the peripheral blood of COVID-19 patients. However, little has been attempted to evaluate immunophenotyping of B cells in patients with different clinical courses of COVID-19. Comparing CD45+ hematopoietic cells from 22 healthy controls and 17 COVID-19 patients, the authors found an expansion of CD19+ B cells in COVID-19 patients with a significant increase in CD138+ ASCs among other B cell subpopulations: transitional, naive, double-negative (DN), and memory [82]. Interestingly, a greater abundance of these mature, CD138+ ASCs, which are often associated with protection during a vaccine-induced response, were found in COVID-19 patients with worse outcomes [82]. Previously, Jenks et al. described an IgD-CD27-double negative B cell population in flaring systemic lupus erythematosus (SLE) patients characterized as part of an extra-follicular (EF) response [83], which is also elevated in rheumatoid arthritis patients [84]. EF B cell activation is particularly prominent in African American SLE patients, a population disproportionately affected in severe COVID-19. In SLE, the EF pathway of activating naïve B cells leads to a large expansion of autoantibodies-ASCs through the primed B cell precursor which is double-negative for naive (IgD) and memory markers (CD27), as well as lacking the expression of CXCR5 and CD21. The Preprint data support DN cells which lack CXCR5 and CD21 expressions suggesting that the global EF pathway could be prominently engaged in COVID-19 patients [82]. Understanding the role of pathogenic B cells could be crucial for designing immuno-modulatory therapies that target pro-inflammatory or potentially autoimmune phenotypes seen with SARS-CoV-2 infections. Thus, this B cell phenotype might serve as an immunological biomarker for severe SARS-CoV-2 infection at early stages.

### 6.4. B Cell Responses

The kinetics of the antibody response to SARS-CoV-2 are currently elucidated as robust B cell responses. There is an urgent need for both sensitive and specific SARS-CoV-2 serological testing to not only reliably identify all infected individuals regardless of clinical symptoms, but also to determine the percentage of convalescent individuals in the population. Multiple serological assays have been used to characterize antibody responses to coronaviruses. The most commonly used assays are called antibody (Ab) assays, which use binding assays, including enzyme linked immunosorbent assays (ELISA), immunofluorescence assays (IFA), Western blots, hemagglutination inhibition (HAI), complement fixation (CF), and neutralization. Antibodies binding the SARS-CoV-2 internal N protein and the external S glycoprotein are commonly detected. Neutralization assays are considered the gold standard for measuring functional antibody responses by IgG, IgM and IgA [85,86]. Similar to SARS-CoV-1 infection, CoV-2 Abs are found in most COVID-19 patients [87]. IgM and then IgG appeared with a median time of 12 and 14 days, respectively. After 8 days, the sensitivity of the Ab assay was higher and reached up to 90% at day 13 and at a later time point, after 15 days, reached 100%, with antibody titers persisting in the weeks following virus clearance [88,89]. There was a strong positive correlation between clinical severity and antibody titer from 2 weeks after illness onset. The high virus-specific antibody titers to SARS-CoV-2 correlated with in-vitro neutralization of the virus and decline in the viral load in patients [90]. The total serum IgM and IgG can be used to determine the level of humoral immune response in COVID-19 patients [50,91].

Antibodies binding to the RBD can be potently neutralizing (nAbs), preventing it from binding to the host entry receptor, ACE2 [89,92]. Anti-RBD nAbs are detected in most COVID-19 patients but no cross reactivity was found to the RBD from SARS-CoV-1 or MERS-CoV and plasma from COVID-19 patients did not neutralize SARS-CoV-1 or MERS-CoV [50,92]. Sequences from single cell sorted RBD-specific CD19+IgG+ memory B cells showed a diverse repertoire with low or no somatic hypermutation. Premkumar et al. generated the recombinant RBDs of SARS-CoV-1, SARS-CoV-2 and human endemic coronaviruses (hCoV HKU-1, OC-43, NL63 and 229E). Their data corroborate previous reports and observations about enhanced specificity of the SARS-CoV-2 induced Abs to its RBD over the RBDs of other CoVs. This suggests pre-existing binding Abs against endemic human coronaviruses seem not to cross-react with the SARS-CoV-2 RBD and that titers of anti-RBD binding Abs robustly correlate with nAb levels [93]. Together, these results make obvious that there is virus-specific antibody mediated neutralization and it is driven by binding to epitopes within the RBD. Clearly understanding the antibody responses against SARS-CoV2 is useful in the development of serological tests for the diagnosis of COVID-19 [90].

## 7. Therapeutics

So far, no specific treatment is available for COVID-19 and only symptomatic and supportive treatments are being pursued [3,6]. The main target is control of the disease manifestation using available drugs. Studies to find the best therapeutic approach for the disease and its management continue to increase. The most common therapeutic options for viral infections are either blocking viral entry and replication or promoting immunity of the uninfected population through vaccination. Therefore, the development of effective vaccines and specific antiviral treatments are the main concern of scientists worldwide [14,94,95,96,97,98,99,100,101]. The genome sequence of SARS-CoV-2 was rapidly characterized, enabling researchers to design vaccines and antiviral drugs, isolate neutralizing monoclonal antibodies and use convalescent plasma as therapy.

## 8. Vaccine Development

Since the emergence of SARS-CoV in 2002, several vaccine approaches have been reported but none has been approved yet by the FDA. During the ongoing SARS-CoV-2 pandemic, unprecedented efforts are being made in vaccine development. Just six months into the pandemic, more than 100 potential vaccines are already into different phases of preclinical or clinical trials. Vaccine design has mainly exploited the viral spike protein to induce anti-viral immunity. Several different vaccine platforms are being evaluated, such as whole virus (attenuated or inactivated) [102,103,104], virus-vectored plasmid encoded viral antigens [94,105] and viral proteins [106,107,108,109,110].

Currently, many institutions and companies are involved in developing SARS-CoV-2 vaccines. On July 2020, several vaccine candidates were included by the global COVID-19 vaccine R&D landscape (for review, [111,112]). Some of the most promising candidate clinical trials were PiCoVacc from Sinovac Biotech (Beijing, China) (NCT04456595), INO-4800 from Inovio (San Diego, CA, USA) (NCT04447781) [113], Ad5-nCoV from CanSino Biologics (Tianjin, China) (NCT04313127) [114], mRNA-1273 from Moderna (Cambridge, MA, USA) (NCT04299724) [115], LV-SMENP-DC (NCT04299724) and pathogen-specific aAPC (NCT04276896) from Shenzhen Medical Institute. Additionally, several inactivated vaccine candidates for SARS-CoV-2 have been developed by different companies such as Wuhan Institute of Biological Products (Sinopharm, Wuhan, China), Beijing Institute of Biological Products (Sinopharm), Bharat Biotech (Hyderabad, India) and Medical Biology, Chinese Academy of Medical Sciences. Their vaccines are currently under clinical trials [112].

**Figure 5:**
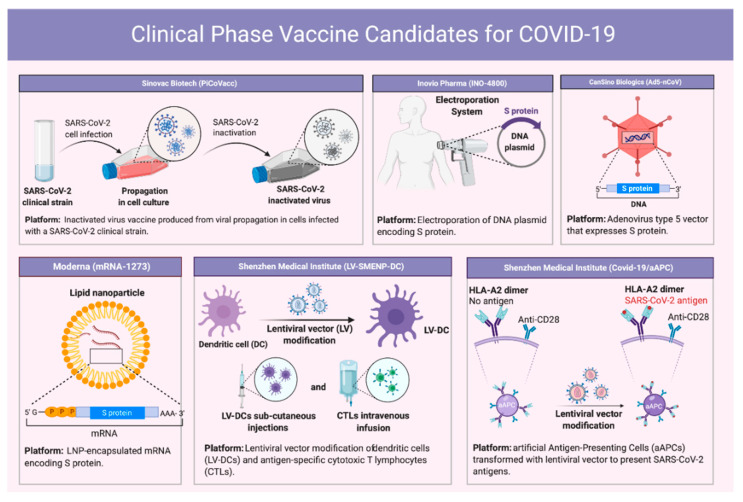
Clinical phase vaccine candidates for COVID-19.

Sinovac Biotech developed a purified inactivated vaccine candidate for SARS-CoV-2 (PiCoVacc) (Figure 5) [116]. In macaques, this vaccine triggered specific neutralizing antibodies and complete protection at a dose of 6 μg without antibody-dependent enhancement (ADE) of infection. These promising results encouraged transition to phase-1 clinical trials. DNA vaccine platforms use plasmids encoding SARS-CoV-2 S protein. This type of vaccine is simple, safe and stable [117]. Inovio Pharma engineered a synthetic DNA vaccine targeting SARS-CoV-2 S protein (INO-4800) similar to their prior vaccine candidate targeting the MERS-CoV S protein (Figure 5) [113]. INO-4800 induced functional antibodies and T cell responses that block SARS-CoV-2 S protein/ACE2 binding. Additionally, CanSino Biologics developed a recombinant adenovirus type-5 (Ad5) vectored COVID-19 vaccine expressing the S protein of SARS-CoV-2 (Ad5-nCoV), which has been shown in a phase 1 trial to be well tolerated and immunogenic (Figure 5) [114]. Currently, it is in a phase II clinical trial. Interestingly, Moderna has designed and developed mRNA-based vaccines encoding the viral S protein (Figure 5) [115]. Their mRNA-1273 is encapsulated in bilipid nanoparticles (LNPs) and has entered phase III clinical trial. Shenzhen Geno-Immune Medical Institute has two vaccine candidates, which have entered phase I studies, LV-SMENP-DC and pathogen-specific aAPC. The LV-SMENP-DC vaccine was formed by modifying dendritic cells (DCs) with lentivirus vectors expressing SMENP (COVID-19 minigene) and immune modulatory genes. LV-DC presenting COVID-19 specific antigens is activated by cytotoxic T lymphocytes (CTLs). COVID-19/aAPC vaccine is also formed from lentivirus modifications with immune-modulatory genes and the viral minigenes to the artificial antigen-presenting cells (aAPCs) [111,112].

## 9. Antiviral Agents against Coronaviruses

Potential antiviral targets can be addressed with an understanding of viral replication and the molecules required for virus pathogenesis. Additionally, the previous experience with SARS-CoV and MERS-CoV epidemics can help in reaching promising treatment strategies against emerging SARS-CoV-2. Repurposing of drugs with known pharmacokinetic profiles is currently being applied to find clinically effective agents against COVID-19. Nucleoside analogues (remdesivir, favipiravir and ribavirin) showed promising in vivo results with SARS-CoVs and MERS-CoVs via interfering synthesis of viral RNA and therefore viral replication [14,94,95,96,97,98,99,100]. Remdesivir (GS-5734) has been extensively tested in humans and showed a reduced recovery time in COVID-19 patients compared to placebo (11 days versus 15 days) [14,94,95,96,97,98,118]. Additionally, corticosteroids such as dexamethasone reduced mortality rates in COVID-19 patients relative to standard care, this is attributed to the broad anti-inflammatory effects of corticosteroids [119].

Protease inhibitors (lopinavir–ritonavir) and antimalarial drugs (chloroquine and its derivative hydroxychloroquine) have been repurposed against COVID-19 because of their potential ability to inhibit the virus replication [94,101,120,121,122,123]. However, the WHO, in July 2020, discontinued the use of these drugs in hospitalized patients due to the non-significant reduction in the mortality, as well as some associated safety issues.

Recently, mesenchymal stem cells were tested against SARS-CoV-2 infection. They can reduce the COVID-19 related cytokine storm induced by the over-activated immune response [46,124].

## 10. Immune Cells and Therapeutic Perspectives

Interferons (IFNs) are a vital component of the innate immune response providing the initial defense against viral infections. IFNs have been extensively used against hepatitis C infections and numerous malignancies [125,126,127,128]. Therefore, IFNs might also be efficient in treating SARS-CoV-2 infection.

Biological agents targeting key pro-inflammatory cytokines, such as interleukin 6 (IL-6), have proved to be effective in the treatment of autoimmune diseases [129]. Anti IL-6 receptor antibody (Tocilizumab) is a humanized recombinant IgG1 monoclonal antibody that binds to the soluble and membrane bound IL-6 receptor [130]. Increased IL-6 is a hallmark of COVID-19 patients along with high levels of C reactive protein, therefore using Tocilizumab has been recommended after effective results in treating such patients to manage cytokine release syndrome (CRS) [129]. Similarly biologics that target IL-1 and IL-17 inflammatory cytokines can also be effectively used in treating COVID-19 patients. IL-1R, GM-CSF, and the chemokine receptor CCR5, have also been proposed as potential blockade targets to manage COVID-19 CRS [50].

## 11. Convalescent Plasma Therapy for COVID-19

Convalescent plasma (CP) may contain therapeutic levels of anti-SARS-CoV-2 neutralizing antibodies, which would reduce subsequent rounds of viral replication and increase viral clearance until we have a vaccine and specific monoclonal antibodies. Passive immunization with CP therapy seems to be associated with improved outcomes and appears to be safe, however, safety and effectiveness for CP transfusion needs to be studied more carefully [43]. Anticoagulant therapy has also been suggested as COVID-19 patients have a higher incidence of venous thromboembolism [131].

## 12. Neutralizing Monoclonal Antibodies

Patients who have recovered from SARS-CoV-2 infection are one potential source of neutralizing antibodies (nAbs) [50,92]. The 193-amino acid receptor-binding domain (RBD) of the spike protein is the key target of neutralizing monoclonal antibodies. Single cell sequencing has been used to clone the heavy and light chain variable regions to express recombinant antibodies from RBD-specific memory B cells. Four of the antibodies produced in these studies (31B5, 32D4, P2C-2F6, P2C-1F11) showed high neutralizing potential in vitro [50]. SARS-CoV-1 and SARS-CoV-2 share about 80% of their sequence identity. Animal models have also been used to generate nAbs against SARS-CoV-2; Wang et al. developed a fully human monoclonal antibody (47D11) in H2L2 mice that neutralizes SARS-CoV-2 (and SARS-CoV) in cell culture [132]. Many pharmaceutical companies are utilizing this approach either as monoclonal or pooled set of nAbs to find a broad-spectrum antibody which is the needed for the treatment until we have a vaccine to provide long-term immunity.

## 13. Cellular Therapy

Cellular therapy using NK cells and mesenchymal stem cells (MSCs) might be a therapeutic option. NK cells are important immune cells necessary for antiviral defense [133]. They lyse antibody-coated virus-infected cells via the process of antibody-dependent cellular cytotoxicity (ADCC) [134]. NK cell therapy to enhance immunity is currently a very feasible strategy for the prevention or treatment of COVID-19 pneumonia.

Another promising approach would be MSCs, which have strong anti-inflammatory and immunomodulatory functions. Numerous studies have shown that treatment with MSCs can ameliorate acute/chronic lung injury and ARDS by suppressing the infiltration of immune cells to pulmonary tissues and pro-inflammatory cytokine secretion [135].

## 14. CRISPR/Cas Mediated Therapeutic Options of COVID-19

Diversity of human viral pathogens and their ability to adapt their environment rapidly requires a flexible and rapidly programmable antiviral technique to control them. To this end, the genome editing technique CRISPR/Cas9 could be a potent weapon against a diverse range of viral pathogens. It could be used to treat diseases caused by viral pathogens by: (i). manipulating host immunity against the pathogen, (ii). destroying the pathogen directly without using immune cells. CRISPR/Cas9 has already been used successfully to reprogram B and T cells for immunotherapy of cancer patients [136] and holds great promise in immunotherapy. Here, we shall shed light on the possibility of using this technique to produce designer immune cells with antiviral activity and an option of using CRISPR to fend off pathogens directly.

## 15. Designer B and T Cells for Antiviral Activity

B cells are a vital component of adaptive immunity in humans and an attractive genome editing target due to their unique function of antibody production in the body [137]. Moreover, easy isolation and cultivation of primary B cells are additional advantages for genome modification for subsequent immunotherapy of diseases [138]. Johnson et al. engineered primary B cells using CRISPR/Cas9 protein to knockout endogenous CD19 genetically and its protein and also integrated a splice acceptor-Enhanced Green Fluorescent Protein (EGFP) cassette into the Adeno-associated virus (AAV) S1 locus using CRISPR/Cas9 protein in combination with AAV serotype 6 (AAV6), an efficient DNA template donor [139]. Their research work indicates that the primary B cells can be engineered to produce specific antibodies against a pathogen. After obtaining mature plasma cells from engineered B cells in vitro, these cells can be reintroduced into the host for long-term high titer expression of antibodies in serum against a specific antigen.

Toll-like receptors (TLRs) in B cells, which belong to the germ line-encoded pattern recognition receptors (PRRs) family, are known to be the most important initiators of an antiviral response upon infection [140]. Among different members of this receptor family, which recognizes different pathogen-associated molecular patterns, TLR7 recognizes many RNA viruses including influenza A virus (IAV) [141]. Moreover, TLR7-mediated humoral immune responses are found to be sufficient in causing two virus strains, mouse mammary tumor virus (MMTV; a betaretrovirus) and murine leukemia virus (MuLV; a gammaretrovirus), to be cleared from mice [142]. Combining the activation of TLR7 with the Johnson et al. findings of producing specific antibodies against pathogens [139], B cells engineered in CRISPR/Cas9, designer B cells against RNA based SARS-CoV-2 virus could be used as a produced for immunotherapy of COVID-19 patients.

Another potent component of the immune system in the body is T cells, which have been engineered using CRISPR/Cas9 for immunotherapy of cancer cells. CRISPR/Cas9 has been used to generate chimeric antigen receptor (CAR) T cells. CAR T-cells are modified T cells that harbor a tumor targeting receptor for a specific antigen on tumor cells. Tumor targeting receptor consists of two components, i.e., an extracellular single chain variable fragment (ScFv) to recognize tumor antigen and an intracellular chimeric signaling domain that activates T cells to kill tumor cells [143]. CAR T-cells have been used successful for B cell malignancy therapy by targeting CD19 antigen expressed by B-cells. Moreover, CAR T-cell based therapies are under evaluation in multiple clinical trials against different solid tumor antigens such as Her2/neu, CEA, EGFR, interleukin-13 receptor alpha 2 (IL13Rα2), MesothelincMet and GD2 [144]. Therefore, it may be possible to design T cell modifications using CRISPR/Cas9 to produce CAR T-cells specific to ssRNA viral pathogens. A specific antigen receptor for COVID-19 can be introduced in T-cells, which will make programmed T-cells more active, resistant to inhibition and exhaustion against SARS-CoV-2 infection in contrast to normal T-cells, which would reduce in number of infected cells during COVID-19 [45].

## 16. Diagnostic and Antiviral Potential of CRISPR/Cas13

CRISPR/Cas technology can not only be utilized for the programming of immune cells but for direct antiviral activity as well. Interestingly, Freije et al. [145] demonstrated strong antiviral activity of a CRISPR/Cas13 against three single stranded RNA viruses: influenza A virus (IAV), vesicular stomatitis virus (VSV) and lymphocytic choriomeningitis virus (LCMV). They have detected several Cas 13 (RNA targeting protein) crRNA target sites in hundreds of ssRNA viral species that normally infect humans and developed a Cas13-based diagnostic and viral RNA destroying tool called CARVER (Cas13-assisted restriction of viral expression and readout). Since CARVER is a reprogrammable diagnostic and viral RNA targeting tool, it has unlimited potential for use in rapid diagnostics for SARS-CoV-2 in human samples by synthesizing crRNA complementary to unique genomic regions of this virus.

## 17. Conclusions

The COVID-19 pandemic has drastically affected global healthcare systems and the worldwide economy. It is the largest pandemic outbreak since the Spanish flu in 1918 and has forced changes in the lifestyles of people in terms of more hygiene and restricted social interactions. Scientists around the world are engaged in research towards finding an efficient disease treatment and vaccine to prevent this fast-spreading disease. Several strategies are already being tried clinically to handle the infection, but still there is a desperate need for effective treatments and vaccines against COVID-19. Multiple potential vaccines have been developed and are in different phases of development with the hope of having a successful vaccine by the end of 2020. Many antiviral drugs, such as remdesivir or lopinavir, are currently used either alone or in combination for COVID-19 treatment in multiple clinical trials. Immunomodulator drugs targeting cytokines are being used to control the cytokine storm. Several viral and immunological parameters are being explored to identify a potential target for cure strategies. Cellular, as well as humoral immune, responses are being targeted by potential vaccines. Consistently, key steps in viral replication are being identified and exploited as pharmacological targets. Therefore, a better understanding of virological, immunological, and clinical details would open avenues for developing potent solutions.

## References

[1] Amanat F., Krammer F. (2020). SARS-CoV-2 Vaccines: Status Report. Immunity.

[2] Petrosillo N., Viceconte G., Ergonul O., Ippolito G., Petersen E. (2020). COVID-19, SARS and MERS: Are they closely related?. Clin. Microbiol. Infect..

[3] Coleman C.M., Frieman M.B. (2014). Coronaviruses: Important emerging human pathogens. J. Virol..

[4] Li W., Shi Z., Yu M., Ren W., Smith C., Epstein J.H., Wang H., Crameri G., Hu Z., Zhang H. (2005). Bats Are Natural Reservoirs of SARS-Like Coronaviruses. Science.

[5] Zaki A.M., van Boheemen S., Bestebroer T.M., Osterhaus A.D.M.E., Fouchier R.A.M. (2012). Isolation of a Novel Coronavirus from a Man with Pneumonia in Saudi Arabia. N. Engl. J. Med..

[6] Udugama B., Kadhiresan P., Kozlowski H.N., Malekjahani A., Osborne M., Li V.Y.C., Chen H., Mubareka S., Gubbay J.B., Chan W.C.W. (2020). Diagnosing COVID-19: The Disease and Tools for Detection. ACS Nano.

[7] NBIC+ An Overview of Nanotechnology Patents Focusing on Coronaviruses. https://statnano.com/news/67513/An-Overview-of-Nanotechnology-Patents-Focusing-on-Coronaviruses.

[8] Lu R., Zhao X., Li J., Niu P., Yang B., Wu H., Wang W., Song H., Huang B., Zhu N. (2020). Genomic characterisation and epidemiology of 2019 novel coronavirus: Implications for virus origins and receptor binding. Lancet.

[9] WHO WHO Announces COVID-19 Outbreak a Pandemic. http://www.euro.who.int/en/health-topics/health-emergencies/coronavirus-covid-19/news/news/2020/3/who-announces-covid-19-outbreak-a-pandemic.

[10] World Health Organization Coronavirus Disease (COVID-19) Situation Report—188. https://www.who.int/docs/default-source/coronaviruse/situation-reports/20200726-covid-19-sitrep-188.pdf?sfvrsn=f177c3fa_2.

[11] Guo Y.-R., Cao Q.-D., Hong Z.-S., Tan Y.-Y., Chen S.-D., Jin H.-J., Tan K.-S., Wang D.-Y., Yan Y. (2020). The origin, transmission and clinical therapies on coronavirus disease 2019 (COVID-19) outbreak–an update on the status. Mil. Med. Res..

[12] Zhu N., Zhang D., Wang W., Li X., Yang B., Song J., Zhao X., Huang B., Shi W., Lu R. (2020). A novel coronavirus from patients with pneumonia in China, 2019. N. Engl. J. Med..

[13] Yin Y., Wunderink R.G. (2018). MERS, SARS and other coronaviruses as causes of pneumonia. Respirology.

[14] Cascella M., Rajnik M., Cuomo A., Dulebohn S.C., Di Napoli R. (2020). Features, evaluation and treatment coronavirus (COVID-19). Statpearls [Internet].

[15] Chen Y., Liu Q., Guo D. (2020). Emerging coronaviruses: Genome structure, replication, and pathogenesis. J. Med. Virol..

[16] Zhou P., Yang X.-L., Wang X.-G., Hu B., Zhang L., Zhang W., Si H.-R., Zhu Y., Li B., Huang C.-L. (2020). A pneumonia outbreak associated with a new coronavirus of probable bat origin. Nature.

[17] Ahn D.-G., Shin H.-J., Kim M.-H., Lee S., Kim H.-S., Myoung J., Kim B.-T., Kim S.-J. (2020). Current Status of Epidemiology, Diagnosis, Therapeutics, and Vaccines for Novel Coronavirus Disease 2019 (COVID-19). J. Microbiol. Biotechnol..

[18] Łoczechin A., Séron K., Barras A., Giovanelli E., Belouzard S., Chen Y.-T., Metzler-Nolte N., Boukherroub R., Dubuisson J., Szunerits S. (2019). Functional Carbon Quantum Dots as Medical Countermeasures to Human Coronavirus. ACS Appl. Mater. Interfaces.

[19] Library D.o.C.-P.H.I. Centers for Disease Control and Prevention. https://phil.cdc.gov/Details.aspx?pid=23354.

[20] Wu A., Peng Y., Huang B., Ding X., Wang X., Niu P., Meng J., Zhu Z., Zhang Z., Wang J. (2020). Genome composition and divergence of the novel coronavirus (2019-nCoV) originating in China. Cell Host Microbe.

[21] Abd Ellah N.H., Gad S.F., Muhammad K., E Batiha G., Hetta H.F. (2020). Nanomedicine as a promising approach for diagnosis, treatment and prophylaxis against covid-19. Nanomedicine.

[22] Wu F., Zhao S., Yu B., Chen Y.-M., Wang W., Song Z.-G., Hu Y., Tao Z.-W., Tian J.-H., Pei Y.-Y. (2020). A new coronavirus associated with human respiratory disease in China. Nature.

[23] Khailany R.A., Safdar M., Ozaslan M. (2020). Genomic characterization of a novel SARS-CoV-2. Gene Rep..

[24] Fehr A.R., Perlman S., Maier H., Bickerton E., Britton P. (2015). Coronaviruses: An overview of their replication and pathogenesis. Coronaviruses.

[25] Bárcena M., Oostergetel G.T., Bartelink W., Faas F.G.A., Verkleij A., Rottier P.J.M., Koster A.J., Bosch B.J. (2009). Cryo-electron tomography of mouse hepatitis virus: Insights into the structure of the coronavirion. Proc. Natl. Acad. Sci. USA.

[26] Neuman B.W., Adair B.D., Yoshioka C., Quispe J.D., Orca G., Kuhn P., Milligan R.A., Yeager M., Buchmeier M.J. (2006). Supramolecular architecture of severe acute respiratory syndrome coronavirus revealed by electron cryomicroscopy. J. Virol..

[27] Huang X., Li M., Xu Y., Zhang J., Meng X., An X., Sun L., Guo L., Shan X., Ge J. (2019). Novel Gold Nanorod-Based HR1 Peptide Inhibitor for Middle East Respiratory Syndrome Coronavirus. ACS Appl. Mater. Interfaces.

[28] Xia S., Zhu Y., Liu M., Lan Q., Xu W., Wu Y., Ying T., Liu S., Shi Z., Jiang S. (2020). Fusion mechanism of 2019-nCoV and fusion inhibitors targeting HR1 domain in spike protein. Cell. Mol. Immunol..

[29] Yu F., Du L., Ojcius D.M., Pan C., Jiang S. (2020). Measures for diagnosing and treating infections by a novel coronavirus responsible for a pneumonia outbreak originating in Wuhan, China. Microbes Infect..

[30] Sawicki S., Sawicki D. (2005). Coronavirus transcription: A perspective. Coronavirus Replication and Reverse Genetics.

[31] Raj V.S., Mou H., Smits S.L., Dekkers D.H.W., Müller M.A., Dijkman R., Muth D., Demmers J.A.A., Zaki A., Fouchier R.A.M. (2013). Dipeptidyl peptidase 4 is a functional receptor for the emerging human coronavirus-EMC. Nature.

[32] Li W., Moore M.J., Vasilieva N., Sui J., Wong S.K., Berne M.A., Somasundaran M., Sullivan J.L., Luzuriaga K., Greenough T.C. (2003). Angiotensin-converting enzyme 2 is a functional receptor for the SARS coronavirus. Nature.

[33] Zhou F., Yu T., Du R., Fan G., Liu Y., Liu Z., Xiang J., Wang Y., Song B., Gu X. (2020). Clinical course and risk factors for mortality of adult inpatients with COVID-19 in Wuhan, China: A retrospective cohort study. Lancet.

[34] Andersen K.G., Rambaut A., Lipkin W.I., Holmes E.C., Garry R.F. (2020). The proximal origin of SARS-CoV-2. Nat. Med..

[35] Huang C., Wang Y., Li X., Ren L., Zhao J., Hu Y., Zhang L., Fan G., Xu J., Gu X. (2020). Clinical features of patients infected with 2019 novel coronavirus in Wuhan, China. Lancet.

[36] Leng Z., Zhu R., Hou W., Feng Y., Yang Y., Han Q., Shan G., Meng F., Du D., Wang S. (2020). Transplantation of ACE2-mesenchymal stem cells improves the outcome of patients with COVID-19 pneumonia. Aging Dis..

[37] McGonagle D., O’Donnell J.S., Sharif K., Emery P., Bridgewood C. (2020). Immune mechanisms of pulmonary intravascular coagulopathy in COVID-19 pneumonia. Lancet Rheumatol..

[38] Connors J.M., Levy J.H. (2020). COVID-19 and its implications for thrombosis and anticoagulation. Blood.

[39] Galbadage T., Peterson B.M., Gunasekera R.S. (2020). Does COVID-19 Spread Through Droplets Alone?. Front. Public Health.

[40] Zhang W., Du R.-H., Li B., Zheng X.-S., Yang X.-L., Hu B., Wang Y.-Y., Xiao G.-F., Yan B., Shi Z.-L. (2020). Molecular and serological investigation of 2019-nCoV infected patients: Implication of multiple shedding routes. Emerg. Microbes Infect..

[41] Zhang R., Li Y.A.-O., Zhang A.L., Wang Y.A.-O., Molina M.J. (2020). Identifying airborne transmission as the dominant route for the spread of COVID-19. Proc. Natl. Acad. Sci. USA.

[42] Setti L., Passarini F., De Gennaro G., Barbieri P., Perrone M.G., Borelli M., Palmisani J., Di Gilio A., Piscitelli P., Miani A. (2020). Airborne Transmission Route of COVID-19: Why 2 Meters/6 Feet of Inter-Personal Distance Could Not Be Enough. Int. J. Environ. Res. Public Health.

[43] Harvala H., Robb M., Watkins N., Ijaz S., Dicks S., Patel M., Supasa P., Dejnirattisai W., Liu C., Mongkolsapaya J. (2020). Convalescent plasma therapy for the treatment of patients with COVID-19: Assessment of methods available for antibody detection and their correlation with neutralising antibody levels. MedRxiv.

[44] Oberfeld B., Achanta A., Carpenter K., Chen P., Gilette N.M., Langat P., Said J.T., Schiff A.E., Zhou A.S., Barczak A.K. (2020). SnapShot: COVID-19. Cell.

[45] Xu Z., Shi L., Wang Y., Zhang J., Huang L., Zhang C., Liu S., Zhao P., Liu H., Zhu L. (2020). Pathological findings of COVID-19 associated with acute respiratory distress syndrome. Lancet Respir. Med..

[46] Atluri S., Manchikanti L., Hirsch J.A. (2020). Expanded Umbilical Cord Mesenchymal Stem Cells (UC-MSCs) as a Therapeutic Strategy in Managing Critically Ill COVID-19 Patients: The Case for Compassionate Use. Pain Physician.

[47] Zhao X., Zhang B., Li P., Ma C., Gu J., Hou P., Guo Z., Wu H., Bai Y. (2020). Incidence, clinical characteristics and prognostic factor of patients with COVID-19: A systematic review and meta-analysis. MedRxiv.

[48] Qu R., Ling Y., Zhang Y.H., Wei L.Y., Chen X., Li X.M., Liu X.Y., Liu H.M., Guo Z., Ren H. (2020). Platelet-to-lymphocyte ratio is associated with prognosis in patients with coronavirus disease-19. J. Med. Virol..

[49] Liu Y., Sun W., Guo Y., Chen L., Zhang L., Zhao S., Long D., Yu L. (2020). Association between platelet parameters and mortality in coronavirus disease 2019: Retrospective cohort study. Platelets.

[50] Vabret N., Britton G.J., Gruber C., Hegde S., Kim J., Kuksin M., Levantovsky R., Malle L., Moreira A., Park M.D. (2020). Immunology of COVID-19: Current State of the Science. Immunity.

[51] Zhou Y., Fu B., Zheng X., Wang D., Zhao C., Qi Y., Sun R., Tian Z., Xu X., Wei H. (2020). Aberrant pathogenic GM-CSF^+^ T cells and inflammatory CD14^+^ CD16^+^ monocytes in severe pulmonary syndrome patients of a new coronavirus. BioRxiv.

[52] Wen W., Su W., Tang H., Le W., Zhang X., Zheng Y., Liu X., Xie L., Li J., Ye J. (2020). Immune cell profiling of COVID-19 patients in the recovery stageby single-cell sequencing. Cell Discov..

[53] Liu J., Li S., Liu J., Liang B., Wang X., Wang H., Li W., Tong Q., Yi J., Zhao L. (2020). Longitudinal characteristics of lymphocyte responses and cytokine profiles in the peripheral blood of SARS-CoV-2 infected patients. EBioMedicine.

[54] Guan W.-J., Ni Z.-Y., Hu Y., Liang W.-H., Ou C.-Q., He J.-X., Liu L., Shan H., Lei C.-l., Hui D.S. (2020). Clinical characteristics of coronavirus disease 2019 in China. N. Engl. J. Med..

[55] Takeuchi O., Akira S. (2009). Innate immunity to virus infection. Immunol. Rev..

[56] Zahran A.M., Zahran Z.A.M., El-Badawy O., Abdel-Rahim M.H., Ali W.A., Rayan A., El-Masry M.A., Abozaid M.A., Hetta H.F. (2019). Prognostic impact of toll-like receptors 2 and 4 expression on monocytes in Egyptian patients with hepatocellular carcinoma. Immunol. Res..

[57] Mogensen T.H. (2009). Pathogen recognition and inflammatory signaling in innate immune defenses. Clin. Microbiol. Rev..

[58] Diebold S.S., Kaisho T., Hemmi H., Akira S., Reis e Sousa C. (2004). Innate antiviral responses by means of TLR7-mediated recognition of single-stranded RNA. Science.

[59] McKechnie J.L., Blish C.A. (2020). The Innate Immune System: Fighting on the Front Lines or Fanning the Flames of COVID-19?. Cell Host Microbe.

[60] Li G., Fan Y., Lai Y., Han T., Li Z., Zhou P., Pan P., Wang W., Hu D., Liu X. (2020). Coronavirus infections and immune responses. J. Med. Virol..

[61] Hetta H.F., Mekky M.A., Zahran A.M., Abdel-Malek M.O., Ramadan H.K., Shafik E.A., Abbas W.A., Abbas El-Masry M., Mohamed N.A., Kamel A.A. (2020). Regulatory B Cells and Their Cytokine Profile in HCV-Related Hepatocellular Carcinoma: Association with Regulatory T Cells and Disease Progression. Vaccines.

[62] Barnes B.J., Adrover J.M., Baxter-Stoltzfus A., Borczuk A., Cools-Lartigue J., Crawford J.M., Dassler-Plenker J., Guerci P., Huynh C., Knight J.S. (2020). Targeting potential drivers of COVID-19: Neutrophil extracellular traps. J. Exp. Med..

[63] Tay M.Z., Poh C.M., Renia L., MacAry P.A., Ng L.F.P. (2020). The trinity of COVID-19: Immunity, inflammation and intervention. Nat. Rev. Immunol..

[64] Pison U., Brand M., Joka T., Obertacke U., Bruch J. (1988). Distribution and function of alveolar cells in multiply injured patients with trauma-induced ARDS. Intensive Care Med..

[65] Park W.B., Kwon N.J., Choi S.J., Kang C.K., Choe P.G., Kim J.Y., Yun J., Lee G.W., Seong M.W., Kim N.J. (2020). Virus Isolation from the First Patient with SARS-CoV-2 in Korea. J. Korean Med. Sci..

[66] Cao X. (2020). COVID-19: Immunopathology and its implications for therapy. Nat. Rev. Immunol..

[67] Merad M., Martin J.C. (2020). Pathological inflammation in patients with COVID-19: A key role for monocytes and macrophages. Nat. Rev. Immunol..

[68] Zheng M., Gao Y., Wang G., Song G., Liu S., Sun D., Xu Y., Tian Z. (2020). Functional exhaustion of antiviral lymphocytes in COVID-19 patients. Cell Mol. Immunol..

[69] Yaqinuddin A., Kashir J. (2020). Innate immunity in COVID-19 patients mediated by NKG2A receptors, and potential treatment using Monalizumab, Cholroquine, and antiviral agents. Med. Hypotheses.

[70] Cecere T.E., Todd S.M., Leroith T. (2012). Regulatory T cells in arterivirus and coronavirus infections: Do they protect against disease or enhance it?. Viruses.

[71] Zahran A.M., Nafady-Hego H., Mansor S.G., Abbas W.A., Abdel-Malek M.O., Mekky M.A., Hetta H.F. (2019). Increased frequency and FOXP3 expression of human CD8+ CD25High+ T lymphocytes and its relation to CD4 regulatory T cells in patients with hepatocellular carcinoma. Hum. Immunol..

[72] Hetta H.F., Zahran A.M., Mansor S.G., Abdel-Malek M.O., Mekky M.A., Abbas W.A. (2019). Frequency and Implications of myeloid-derived suppressor cells and lymphocyte subsets in Egyptian patients with hepatitis C virus-related hepatocellular carcinoma. J. Med. Virol..

[73] Grifoni A., Weiskopf D., Ramirez S.I., Mateus J., Dan J.M., Moderbacher C.R., Rawlings S.A., Sutherland A., Premkumar L., Jadi R.S. (2020). Targets of T Cell Responses to SARS-CoV-2 Coronavirus in Humans with COVID-19 Disease and Unexposed Individuals. Cell.

[74] Chen J., Lau Y.F., Lamirande E.W., Paddock C.D., Bartlett J.H., Zaki S.R., Subbarao K. (2010). Cellular immune responses to severe acute respiratory syndrome coronavirus (SARS-CoV) infection in senescent BALB/c mice: CD4+ T cells are important in control of SARS-CoV infection. J. Virol..

[75] Zhao J., Zhao J., Perlman S. (2010). T cell responses are required for protection from clinical disease and for virus clearance in severe acute respiratory syndrome coronavirus-infected mice. J. Virol..

[76] Braun J., Loyal L., Frentsch M., Wendisch D., Georg P., Kurth F., Hippenstiel S., Dingeldey M., Kruse B., Fauchere F. (2020). SARS-CoV-2-reactive T cells in healthy donors and patients with COVID-19. Nature.

[77] Gimenez E., Albert E., Torres I., Remigia M.J., Alcaraz M.J., Galindo M.J., Blasco M.L., Solano C., Forner M.J., Redon J. (2020). SARS-CoV-2-reactive interferon-gamma-producing CD8+ T cells in patients hospitalized with coronavirus disease 2019. J. Med. Virol..

[78] Weiskopf D., Schmitz K.S., Raadsen M.P., Grifoni A., Okba N.M.A., Endeman H., van den Akker J.P.C., Molenkamp R., Koopmans M.P.G., van Gorp E.C.M. (2020). Phenotype and kinetics of SARS-CoV-2–specific T cells in COVID-19 patients with acute respiratory distress syndrome. Sci. Immunol..

[79] Le Bert N., Tan A.T., Kunasegaran K., Tham C.Y.L., Hafezi M., Chia A., Chng M.H.Y., Lin M., Tan N., Linster M. (2020). SARS-CoV-2-specific T cell immunity in cases of COVID-19 and SARS, and uninfected controls. Nature.

[80] Hetta H.F., Mwafey I.M., Batiha G.E.-S., Alomar S.Y., Mohamed N.A., Ibrahim M.A., Elkady A., Meshaal A.K., Alrefai H., Khodeer D.M. (2020). Cd19+ cd24hi cd38hi regulatory b cells and memory b cells in periodontitis: Association with pro-inflammatory and anti-inflammatory cytokines. Vaccines.

[81] Palm A.-K.E., Henry C. (2019). Remembrance of Things Past: Long-Term B Cell Memory After Infection and Vaccination. Front. Immunol..

[82] Woodruff M., Ramonell R., Cashman K., Nguyen D., Ley A., Kyu S., Saini A., Haddad N., Chen W., Howell J.C. (2020). Critically ill SARS-CoV-2 patients display lupus-like hallmarks of extrafollicular B cell activation. MedRxiv.

[83] Jenks S.A., Cashman K.S., Zumaquero E., Marigorta U.M., Patel A.V., Wang X., Tomar D., Woodruff M.C., Simon Z., Bugrovsky R. (2018). Distinct Effector B Cells Induced by Unregulated Toll-like Receptor 7 Contribute to Pathogenic Responses in Systemic Lupus Erythematosus. Immunity.

[84] Mahmood Z., Muhammad K., Schmalzing M., Roll P., Dörner T., Tony H.-P. (2015). Cd27-igd- memory b cells are modulated by in vivo interleukin-6 receptor (il-6r) blockade in rheumatoid arthritis. Arthritis Res. Ther..

[85] Huang A.T., Garcia-Carreras B., Hitchings M.D.T., Yang B., Katzelnick L., Rattigan S.M., Borgert B., Moreno C., Solomon B.D., Rodriguez-Barraquer I. (2020). A systematic review of antibody mediated immunity to coronaviruses: Antibody kinetics, correlates of protection, and association of antibody responses with severity of disease. MedRxiv.

[86] Amanat F., Stadlbauer D., Strohmeier S., Nguyen T., Chromikova V., McMahon M., Jiang K., Asthagiri-Arunkumar G., Jurczyszak D., Polanco J. (2020). A serological assay to detect SARS-CoV-2 seroconversion in humans. MedRxiv.

[87] Hsueh P.R., Huang L.M., Chen P.J., Kao C.L., Yang P.C. (2004). Chronological evolution of IgM, IgA, IgG and neutralisation antibodies after infection with SARS-associated coronavirus. Clin. Microbiol. Infect..

[88] Haveri A., Smura T., Kuivanen S., Österlund P., Hepojoki J., Ikonen N., Pitkäpaasi M., Blomqvist S., Rönkkö E., Kantele A. (2020). Serological and molecular findings during SARS-CoV-2 infection: The first case study in Finland, January to February 2020. Eurosurveillance.

[89] Wu F., Wang A., Liu M., Wang Q., Chen J., Xia S., Ling Y., Zhang Y., Xun J., Lu L. (2020). Neutralizing antibody responses to SARS-CoV-2 in a COVID-19 recovered patient cohort and their implications. MedRxiv.

[90] Lou B., Li T., Zheng S., Su Y., Li Z., Liu W., Yu F., Ge S., Zou Q., Yuan Q. (2020). Serology characteristics of SARS-CoV-2 infection since the exposure and post symptoms onset. MedRxiv.

[91] Zhao J., Yuan Q., Wang H., Liu W., Liao X., Su Y., Wang X., Yuan J., Li T., Li J. (2020). Antibody responses to SARS-CoV-2 in patients of novel coronavirus disease 2019. MedRxiv.

[92] Ju B., Zhang Q., Ge J., Wang R., Sun J., Ge X., Yu J., Shan S., Zhou B., Song S. (2020). Human neutralizing antibodies elicited by SARS-CoV-2 infection. Nature.

[93] Premkumar L., Segovia-Chumbez B., Jadi R., Martinez D.R., Raut R., Markmann A., Cornaby C., Bartelt L., Weiss S., Park Y. (2020). The RBD Of The Spike Protein Of SARS-Group Coronaviruses Is A Highly Specific Target Of SARS-CoV-2 Antibodies But Not Other Pathogenic Human and Animal Coronavirus Antibodies. MedRxiv.

[94] Wang M., Cao R., Zhang L., Yang X., Liu J., Xu M., Shi Z., Hu Z., Zhong W., Xiao G. (2020). Remdesivir and chloroquine effectively inhibit the recently emerged novel coronavirus (2019-nCoV) in vitro. Cell Res..

[95] Holshue M.L., DeBolt C., Lindquist S., Lofy K.H., Wiesman J., Bruce H., Spitters C., Ericson K., Wilkerson S., Tural A. (2020). First Case of 2019 Novel Coronavirus in the United States. N. Engl. J. Med..

[96] Reina J. (2020). Remdesivir, the antiviral hope against SARS-CoV-2. Rev. Esp. Quimioter..

[97] Sheahan T.P., Sims A.C., Leist S.R., Schäfer A., Won J., Brown A.J., Montgomery S.A., Hogg A., Babusis D., Clarke M.O. (2020). Comparative therapeutic efficacy of remdesivir and combination lopinavir, ritonavir, and interferon beta against MERS-CoV. Nat. Commun..

[98] Kruse R.L. (2020). Therapeutic strategies in an outbreak scenario to treat the novel coronavirus originating in Wuhan, China. F1000Research.

[99] Zumla A., Chan J.F.W., Azhar E.I., Hui D.S.C., Yuen K.-Y. (2016). Coronaviruses—Drug discovery and therapeutic options. Nat. Rev. Drug Discov..

[100] Al-Tawfiq J.A., Momattin H., Dib J., Memish Z.A. (2014). Ribavirin and interferon therapy in patients infected with the Middle East respiratory syndrome coronavirus: An observational study. Int. J. Infect. Dis..

[101] Vincent M.J., Bergeron E., Benjannet S., Erickson B.R., Rollin P.E., Ksiazek T.G., Seidah N.G., Nichol S.T. (2005). Chloroquine is a potent inhibitor of SARS coronavirus infection and spread. Virol. J..

[102] Menachery V.D., Gralinski L.E., Mitchell H.D., Dinnon K.H., Leist S.R., Yount B.L., McAnarney E.T., Graham R.L., Waters K.M., Baric R.S. (2018). Combination attenuation offers strategy for live attenuated coronavirus vaccines. J. Virol..

[103] Graham R.L., Deming D.J., Deming M.E., Yount B.L., Baric R.S. (2018). Evaluation of a recombination-resistant coronavirus as a broadly applicable, rapidly implementable vaccine platform. Commun. Biol..

[104] Spruth M., Kistner O., Savidis-Dacho H., Hitter E., Crowe B., Gerencer M., Brühl P., Grillberger L., Reiter M., Tauer C. (2006). A double-inactivated whole virus candidate SARS coronavirus vaccine stimulates neutralising and protective antibody responses. Vaccine.

[105] Wang S.-F., Tseng S.-P., Yen C.-H., Yang J.-Y., Tsao C.-H., Shen C.-W., Chen K.-H., Liu F.-T., Liu W.-T., Chen Y.-M.A. (2014). Antibody-dependent SARS coronavirus infection is mediated by antibodies against spike proteins. Biochem. Biophys. Res. Commun..

[106] Graham R.L., Donaldson E.F., Baric R.S. (2013). A decade after SARS: Strategies for controlling emerging coronaviruses. Nat. Rev. Microbiol..

[107] Okba N.M.A., Raj V.S., Haagmans B.L. (2017). Middle East respiratory syndrome coronavirus vaccines: Current status and novel approaches. Curr. Opin. Virol..

[108] Sui J., Li W., Murakami A., Tamin A., Matthews L.J., Wong S.K., Moore M.J., Tallarico A.S.C., Olurinde M., Choe H. (2004). Potent neutralization of severe acute respiratory syndrome (SARS) coronavirus by a human mAb to S1 protein that blocks receptor association. Proc. Natl. Acad. Sci. USA.

[109] Du L., He Y., Zhou Y., Liu S., Zheng B.-J., Jiang S. (2009). The spike protein of SARS-CoV—A target for vaccine and therapeutic development. Nat. Rev. Microbiol..

[110] Zakhartchouk A.N., Sharon C., Satkunarajah M., Auperin T., Viswanathan S., Mutwiri G., Petric M., See R.H., Brunham R.C., Finlay B.B. (2007). Immunogenicity of a receptor-binding domain of SARS coronavirus spike protein in mice: Implications for a subunit vaccine. Vaccine.

[111] Le T.T., Andreadakis Z., Kumar A., Roman R.G., Tollefsen S., Saville M., Mayhew S. (2020). The COVID-19 vaccine development landscape. Nat. Rev. Drug Discov..

[112] World Health Organization Draft Landscape of COVID-19 Candidate Vaccines. https://www.who.int/publications/m/item/draft-landscape-of-covid-19-candidate-vaccines.

[113] Smith T.R.F., Patel A., Ramos S., Elwood D., Zhu X., Yan J., Gary E.N., Walker S.N., Schultheis K., Purwar M. (2020). Immunogenicity of a DNA vaccine candidate for COVID-19. Nat. Commun..

[114] Zhu F.-C., Li Y.-H., Guan X.-H., Hou L.-H., Wang W.-J., Li J.-X., Wu S.-P., Wang B.-S., Wang Z., Wang L. (2020). Safety, tolerability, and immunogenicity of a recombinant adenovirus type-5 vectored COVID-19 vaccine: A dose-escalation, open-label, non-randomised, first-in-human trial. Lancet.

[115] Wang F., Kream R.M., Stefano G.B. (2020). An Evidence Based Perspective on mRNA-SARS-CoV-2 Vaccine Development. Med. Sci. Monit..

[116] Gao Q., Bao L., Mao H., Wang L., Xu K., Yang M., Li Y., Zhu L., Wang N., Lv Z. (2020). Development of an inactivated vaccine candidate for SARS-CoV-2. Science.

[117] Kim T.W., Lee J.H., Hung C.-F., Peng S., Roden R., Wang M.-C., Viscidi R., Tsai Y.-C., He L., Chen P.-J. (2004). Generation and characterization of DNA vaccines targeting the nucleocapsid protein of severe acute respiratory syndrome coronavirus. J. Virol..

[118] Beigel J.H., Tomashek K.M., Dodd L.E., Mehta A.K., Zingman B.S., Kalil A.C., Hohmann E., Chu H.Y., Luetkemeyer A., Kline S. (2020). Remdesivir for the Treatment of Covid-19—Preliminary Report. N. Engl. J. Med..

[119] Johnson R.M., Vinetz J.M. (2020). Dexamethasone in the management of covid-19. BMJ.

[120] Wu C.-Y., Jan J.-T., Ma S.-H., Kuo C.-J., Juan H.-F., Cheng Y.-S.E., Hsu H.-H., Huang H.-C., Wu D., Brik A. (2004). Small molecules targeting severe acute respiratory syndrome human coronavirus. Proc. Natl. Acad. Sci. USA.

[121] Chu C.M., Cheng V.C.C., Hung I.F.N., Wong M.M.L., Chan K.H., Chan K.S., Kao R.Y.T., Poon L.L.M., Wong C.L.P., Guan Y. (2004). Role of lopinavir/ritonavir in the treatment of SARS: Initial virological and clinical findings. Thorax.

[122] Cao B., Wang Y., Wen D., Liu W., Wang J., Fan G., Ruan L., Song B., Cai Y., Wei M. (2020). A Trial of Lopinavir–Ritonavir in Adults Hospitalized with Severe Covid-19. N. Engl. J. Med..

[123] World Health Organization “Solidarity” Clinical Trial for COVID-19 Treatments. https://www.who.int/emergencies/diseases/novel-coronavirus-2019/global-research-on-novel-coronavirus-2019-ncov/solidarity-clinical-trial-for-covid-19-treatments.

[124] Rajarshi K., Chatterjee A., Ray S. (2020). Combating COVID-19 with mesenchymal stem cell therapy. Biotechnol. Rep..

[125] Borden E.C., Sen G.C., Uze G., Silverman R.H., Ransohoff R.M., Foster G.R., Stark G.R. (2007). Interferons at age 50: Past, current and future impact on biomedicine. Nat. Rev. Drug Discov..

[126] Hetta H.F., Mekky M.A., Khalil N.K., Mohamed W.A., El-Feky M.A., Ahmed S.H., Daef E.A., Medhat A., Nassar M.I., Sherman K.E. (2016). Extra-hepatic infection of hepatitis C virus in the colon tissue and its relationship with hepatitis C virus pathogenesis. J. Med. Microbiol..

[127] Hetta H.F., Mekky M.A., Khalil N.K., Mohamed W.A., El-Feky M.A., Ahmed S.H., Daef E.A., Nassar M.I., Medhat A., Sherman K.E. (2015). Association of colonic regulatory T cells with hepatitis C virus pathogenesis and liver pathology. J. Gastroenterol. Hepatol..

[128] Mehta M., Hetta H.F., Abdel-Hameed E.A., Rouster S.D., Hossain M., Mekky M.A., Khalil N.K., Mohamed W.A., El-Feky M.A., Ahmed S.H. (2016). Association between IL28b rs12979860 single nucleotide polymorphism and the frequency of colonic T reg in chronically HCV-infected patients. Arch. Virol..

[129] Choy E.H., De Benedetti F., Takeuchi T., Hashizume M., John M.R., Kishimoto T. (2020). Translating IL-6 biology into effective treatments. Nat. Rev. Rheumatol..

[130] Roll P., Muhammad K., Schumann M., Kleinert S., Einsele H., Dorner T., Tony H.P. (2011). In vivo effects of the anti-interleukin-6 receptor inhibitor tocilizumab on the B cell compartment. Arthritis Rheum..

[131] Kollias A., Kyriakoulis K.G., Dimakakos E., Poulakou G., Stergiou G.S., Syrigos K. (2020). Thromboembolic risk and anticoagulant therapy in COVID-19 patients: Emerging evidence and call for action. Br. J. Haematol..

[132] Wang D., Hu B., Hu C., Zhu F., Liu X., Zhang J., Wang B., Xiang H., Cheng Z., Xiong Y. (2020). Clinical Characteristics of 138 Hospitalized Patients With 2019 Novel Coronavirus-Infected Pneumonia in Wuhan, China. JAMA.

[133] Zahran A.M., Ashmawy A.M., Rayan A., Elkady A., Elsherbiny N.M., Hetta H.F. (2018). Frequency and implications of natural killer and natural killer T cells in hepatocellular carcinoma. Egypt. J. Immunol..

[134] Hammer Q., Ruckert T., Romagnani C. (2018). Natural killer cell specificity for viral infections. Nat. Immunol..

[135] Li H., Liu S.-M., Yu X.-H., Tang S.-L., Tang C.-K. (2020). Coronavirus disease 2019 (COVID-19): Current status and future perspectives. Int. J. Antimicrob. Agents.

[136] Liu D., Chen M., Mendoza B., Cheng H., Hu R., Li L., Trinh C.T., Tuskan G.A., Yang X. (2019). CRISPR/Cas9-mediated targeted mutagenesis for functional genomics research of crassulacean acid metabolism plants. J. Exp. Bot..

[137] Dorner T., Radbruch A. (2007). Antibodies and B cell memory in viral immunity. Immunity.

[138] Liebig T.M., Fiedler A., Zoghi S., Shimabukuro-Vornhagen A., von Bergwelt-Baildon M.S. (2009). Generation of human CD40-activated B cells. J. Vis. Exp..

[139] Johnson M.J., Laoharawee K., Lahr W.S., Webber B.R., Moriarity B.S. (2018). Engineering of Primary Human B cells with CRISPR/Cas9 Targeted Nuclease. Sci. Rep..

[140] Akira S., Uematsu S., Takeuchi O. (2006). Pathogen recognition and innate immunity. Cell.

[141] Jensen S., Thomsen A.R. (2012). Sensing of RNA viruses: A review of innate immune receptors involved in recognizing RNA virus invasion. J. Virol..

[142] Kane M., Case L.K., Wang C., Yurkovetskiy L., Dikiy S., Golovkina T.V. (2011). Innate immune sensing of retroviral infection via Toll-like receptor 7 occurs upon viral entry. Immunity.

[143] Carpenito C., Milone M.C., Hassan R., Simonet J.C., Lakhal M., Suhoski M.M., Varela-Rohena A., Haines K.M., Heitjan D.F., Albelda S.M. (2009). Control of large, established tumor xenografts with genetically retargeted human T cells containing CD28 and CD137 domains. Proc. Natl. Acad. Sci. USA.

[144] Ren J., Zhao Y. (2017). Advancing chimeric antigen receptor T cell therapy with CRISPR/Cas9. Protein Cell.

[145] Freije C.A., Myhrvold C., Boehm C.K., Lin A.E., Welch N.L., Carter A., Metsky H.C., Luo C.Y., Abudayyeh O.O., Gootenberg J.S. (2019). Programmable Inhibition and Detection of RNA Viruses Using Cas13. Mol. Cell.

